# MgO/KH_2_PO_4_ and Curing Moisture Content in MKPC Matrices to Optimize the Immobilization of Pure Al and Al-Mg Alloys

**DOI:** 10.3390/ma17061263

**Published:** 2024-03-08

**Authors:** Carla Fernández-García, María Cruz Alonso, José María Bastidas, Inés García-Lodeiro, Raúl Fernández

**Affiliations:** 1 Institute of Construction Science “Eduardo Torroja” (IETcc), CSIC, Serrano Galvache 4, 28033 Madrid, Spain; carla.fernandez@ietcc.csic.es (C.F.-G.); iglodeiro@ietcc.csic.es (I.G.-L.); 2 National Centre for Metallurgical Research (CENIM), CSIC, Ave. Gregorio Del Amo 8, 28040 Madrid, Spain; bastidas@cenim.csic.es; 3 Department of Geology and Geochemistry, Faculty of Sciences, Autonomous University of Madrid (UAM), 28049 Madrid, Spain; raul.fernandez@uam.es

**Keywords:** magnesium potassium phosphate cement (MKPC), immobilization of Al radioactive waste, corrosion, hydrogen release, pH, pore ion content

## Abstract

Magnesium Potassium Phosphate Cements (MKPCs) are considered a good alternative for the immobilization of aluminium radioactive waste. MKPC composition and moisture curing conditions are relevant issues to be evaluated. The corrosion of pure aluminium (A1050) and AlMg alloys (AA5754) with 3.5% of Mg is studied in MKPC systems prepared with different MgO/KH_2_PO_4_ (M/P) molar ratios (1, 2, and 3M) and moisture curing conditions (100% Relative Humidity (RH) and isolated in plastic containers (endogenous curing)). The Al corrosion potential (E_corr_) and corrosion kinetic (i_corr_ and V_corr_) are evaluated over 90 days. Additionally, the pore ion evolution, the matrix electrical resistance, the pore structure, and compressive strength are analysed. The corrosion process of Al alloy is affected by the pH and ion content in the pore solution. The pore pH increases from near neutral for the 1M M/P ratio to 9 and 10 for the 2 and 3M M/P ratio, increasing in the same way the corrosion of pure Al (AA1050) and AlMg alloys (AA5754). The effect of Mg content in the alloy (AA5754) becomes more relevant with the increase in the M/P ratio. The presence of phosphate ions in the pore solution inhibits the corrosion process in both Al alloys. The MKPC physicochemical stability improved with the increase in the M/P ratio, higher mechanical strength, and more refined pore structure.

## 1. Introduction

Low- and intermediate-level radioactive solid and liquid wastes (LILW) need to be stabilized before sending to the geological repository. These nuclear wastes are placed into steel containers using a cement matrix for the immobilization of the waste as part of the engineering barrier system [1,2,3]. Ordinary Portland cement (OPC) is commonly used as a cementitious material for the immobilization of these wastes. However, to ensure safe storage, it is necessary to determine the reactivity and the stability of the radioactive waste in the cementitious matrix and in the disposal environment. 

LILW may contain radioactive metals [4] derived from the decommissioning of nuclear power plants and other industrial activities. These metals can chemically interact with the ions dissolved in the pore cementitious matrix, causing instability and compromising the long-term conditioning of the radioactive metal. One critical element for the immobilization of radioactive metals is the pH of the cement pore solution. Metals have a critical pH passivity domain [5] that, in the case of aluminium, covers from pH 4 to 9; for beryllium, from pH 2.9 to 11.7; and for activated magnox metal of magnesium–aluminium alloys, pH > 10.5. These pH critical values must be considered for the immobilization of the respective radioactive metal in the optimal cementitious matrix. According to this, other alternative cementitious matrices to the commonly used OPC are reported in the literature, such as brucite cement for beryllium [6], magnesium phosphate cement for aluminium [7,8], or OPC blended with secondary cementitious materials such as blast furnace slags (BFS) or fly ash (FA) for magnox alloy [9], which have demonstrated good behaviour. 

In this context, the present study focuses on the immobilization of radioactive aluminium in alternative cementitious systems, magnesium phosphate cement (MKPC). Al is an amphoteric metal that reacts in the environment, growing a protective alumina oxide layer (Al_2_O_3_) in contact with air or in water, as described in Equation (1) [5], which is stable in a pH range from 4 to 9, identified as the Al passivation domain [5,10].

2Al + 6H_2_O → Al_2_O_3_∙3H_2_O + 3H_2_ (1)

Outside of this pH range, the Al_2_O_3_ protective layer formed on the surface of the aluminium metal is soluble, and corrosion of the Al metal continues with associated hydrogen gas accumulation according to the redox reaction in an acidic media (pH < 4) (see Equation (2)) and in a basic media (pH > 9) (see (Equation (3)) [5]:

2Al + 6H^+^ → 2Al^3+^ + 3H_2_ (2)

2Al + 2OH^−^ + 2H_2_O → 2AlO_2_^−^ + 3H_2_ (3)

The process in Equation (3) is that expected in OPC matrices with an alkaline nature, contributing to increasing the pressure in the waste metallic container used for the radioactive waste storage system and leading to its cracking and explosion risk.

Due to the high alkalinity of conventional OPC cement matrices (pH > 13), their use to encapsulate radioactive Al is not recommended. MKPC matrices, with almost neutral pore pH, have been proposed as a potential alternative for Al immobilization [11,12]. MKPC, with a magnesia to phosphate molar ratio of 1, is a type of cement that hardens via an acidic–basic reaction between an alkaline magnesia source (MgO) and an acid phosphoric salt, such as potassium dihydrogen phosphate (KH_2_PO_4_) [13] in the presence of water. The main reaction involved in MKPC is summarized in Equation (4):

MgO + KH_2_PO_4_ + 5H_2_O → KMgPO_4_∙6H_2_O (4)

According to Equation (4), magnesium potassium phosphate hexahydrate (KMgPO4·6H_2_O), also known as K-struvite, is the MKPC stoichiometric reaction product. This chemical reaction involves the dissolution of MgO, producing Mg^2+^ and OH^−^ ions, and then the interaction with KH_2_PO_4_ (PO_4_^3−^ and K^+^ ions), resulting in the precipitation of K-struvite. These reactions involve a high exothermic process, and the incorporation of inorganic additions (e.g., FA, metakaolin or pumice) can contribute to decreasing the total heat and improve the chemical and mechanical properties in a more controlled thermal environment [14]. Due to the rapid acidic–basic reaction, a chemical retarder (e.g., orthoboric acid (H_3_BO_3_)) is usually introduced to delay the kinetics of the reaction and control the setting time [15]. MKPC binders with a 1M magnesia-to-phosphate ratio (M/P ratio) have potential advantageous properties with respect to Portland cementitious matrices, such as their lower pH, rapid strength development, resistance to high-temperature environments, low permeability, and excellent corrosion resistance for Al immobilization [16].

However, there are many factors, such as the M/P ratio and the moisture content during curing, that influence the performance of the final MKPC. In the MKPC matrix with near-neutral pH, the aluminium is more stable, thus reducing the risk of Al corrosion and the amount of H_2_ released for long-term management of the radioactive metal. This is the reason why the main criteria for aluminium immobilization are based on the volume of H_2_ production.

Studies in the literature have reported the volume of hydrogen (per unit embedded metal surface) produced by the corrosion of aluminium in OPC and MKPC cement systems employing gas chromatography (GC) [8,17]. Massive amounts of H_2_ gas in OPC cement pastes of about 40 L/m^2^ [8] and 50 L/m^2^ in mortar systems have been reported after one year of exposure [17]. Lower volumes of H_2_ were detected with values of 0.03 L/m^2^ in cement pastes [8] and 0.5 L/m^2^ in mortar [17] for the same period with MKPC systems. Also, H_2_ release values estimated from electrochemical measurements, such as impedance spectroscopy (EIS), have been reported [17] to be about 250 L/m^2^ in OPC mortars and 2 L/m^2^ in MKPC mortars over 250 days of testing. The main differences between both techniques can be due to a reaction inside the cement-based matrix or partial adsorption of H_2_ in the mortar, as explained by [17]. Hydrogen release volumes during the early age of contact (15 days) have been estimated for the Al corrosion rate determined from Linear Polarization Resistance (LPR) [18], with values of 0.14 to 0.33 L/m^2^ in mortars MKPC 1M and 11.6 to 17.8 L/m^2^ in standard OPC mortars for pure Al (A1050) and AlMg (3.5%) (AA5754) alloy, respectively.

The mix design, such as the M/P ratio, and the moisture content during curing play an important role in the physical–chemical stability of MKPC matrices and in the aluminium reactivity. Wang et al. [19] studied the influence of MgO/KH_2_PO_4_ molar ratio of 1 to 5 on the microstructural properties of MKPC cement pastes cured at 100%RH and room temperature and concluded that lower M/P ratios show lower strength to compression. Wang et al. [20] also analysed the effect of increasing the M/P molar ratio (7 to 17) in MKPC cement pastes, working at a constant temperature of 20 °C, and found that the pH of MKPC pore solution increases at a higher M/P ratio, with a decrease in the concentration of phosphate ions in MKPC pore solution. Chong et al. [21] studied the influence of external environmental humidity (air curing, 98%RH, 65 ± 5%RH, and water immersion) on MKPC cement pastes using an M/P mass ratio of 3 and found that air curing results in higher compressive strength and lower total porosity in contrast with the water immersion. Wang et al. [20] also studied the effect of the M/P ratio and curing humidity on the corrosion phenomena of different metallic alloys. For Mg alloy, Wang et al. [20] found that an increase in corrosion resistance in M/P molar ratio from 7 to 17 implies an increase in the pore pH. The increase in corrosion resistance was attributed to the adsorption of phosphate ions on the surface of the Mg alloy oxide film, which consists of magnesium phosphate compounds. Chong et al. [21] also identified a reduction in the corrosion degree of Al-Zn-Mg alloy under air curing, resulting in a lower amount of corrosion products on the metal surface.

To enhance the efficiency of the long-term immobilization of radioactive Al in MKPC matrices, it is necessary to understand the role of the MgO/KH_2_PO_4_ (M/P) ratio and the moisture content at curing that affect the MKPC matrix stability and the corrosion kinetic of Al, which is the main aim of this work. Three M/P ratios and two curing conditions were considered (the standard 100%RH and isolated in plastic containers to simulate the site conditioning in the drum). A pure Al (A1050) and AlMg (AA5754) alloy embedded in MKPC mortars was used to assess the effect of their different electrochemical reactivity to understand the corrosion kinetic and quantify the volume of H_2_ gas released from the Al alloys. Moreover, the understanding of the physical-chemical processes involved in the microstructure and stability of MKPC matrices was determined through analyses of physical, mineralogical, and chemical properties.

## 2. Materials and Methodology

### 2.1. Raw Materials 

Two different grades of commercial aluminium alloy were used, as shown in Table 1.

**Figure 1:**
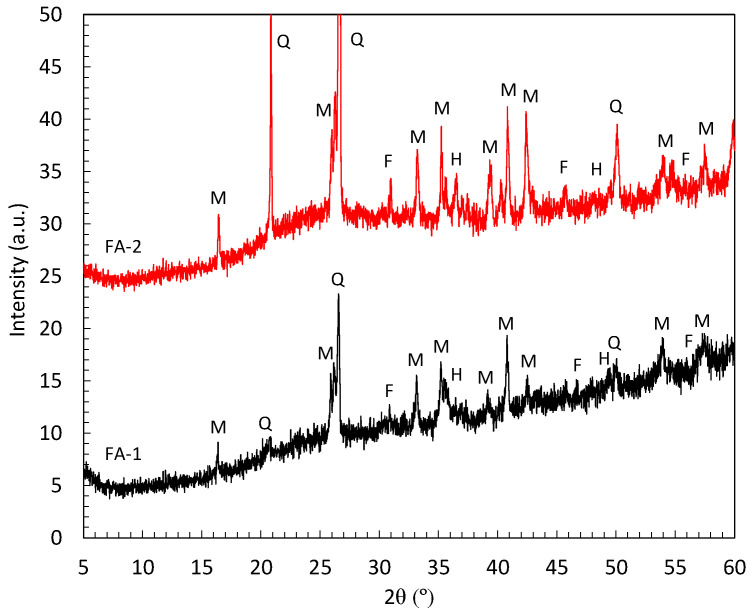
XRD patterns of the FA type F. Legend: M: mullite, Q: quartz, F: magnetite, H: hematite.

Hard-burnt magnesia (MgO, 97.45%) from Martin Marietta Magnesia Specialties, an American-based company and a leading supplier of high-purity magnesia and dolomitic lime products, was used to prepare the MKPC matrices. Chemical composition of MgO product is summarized in Table 2, which presents high crystalline content of periclase as main crystalline phase, as observed in previous studies [18]. A low-cost KH_2_PO_4_ (fertilizer grade of 98%) supplied by Yara (Krista TM) was employed. H_3_BO_3_ (>96%) from VWR Chemicals was added as a retarder to delay the acid–base reaction and control the setting time. FA type F from two different sources was introduced as filler material to enhance the fluidity, limit the temperature rise during the setting time, and control the shrinkage risk. XRD pattern of FA shows (see Figure 1) an amorphous hump associated with the vitreous structure, together with the presence of mullite, quartz, and hematite as secondary phases. 

### 2.2. MKPC Mortar and Cement Paste Preparation 

MKPC mortar and paste samples were prepared using different MgO/KH_2_PO_4_ (M/P) molar ratios of 1, 2, and 3 for matrix characterization tests. For corrosion studies, MKPC mortars with coupons of pure Al (A1050) and AlMg (AA5754) alloys embedded were also prepared for corrosion performance study. Additionally, MKPC mortars, with an M/P 1 molar ratio and two stainless-steel meshes embedded, were used for the characterization of the electrical properties of the MKPC matrix. 

Dosages used in this study are given in Table 3. A water/(MgO + KH_2_PO_4_) mass ratio of 0.5 for mortars and 0.3 for cement pastes were employed. The FA/(MgO + KH_2_PO_4_) and H_3_BO_3_/(MgO + KH_2_PO_4_) mass ratios were 1 and 0.02, respectively, according to [22]. In mortars, a standardized graded sand with 99% silica content was incorporated with a sand/solid mass ratio of 1. Specimens were cured at a temperature of 22 ± 2 °C under two different moisture conditions: (1) in a chamber at 100%RH and (2) isolated in sealed plastic containers, identified as endogenous curing. MKPC samples used in this study are summarized in Table 4.

### 2.3. Characterization Methodology 

#### 2.3.1. MKPC Matrix Characterization

Mechanical strengths were performed according to UNE EN 196 1 standard [23] using an Autest 200 machine of Ibertest (Madrid, Spain). Replicate samples of cement paste samples of 1 × 1 × 6 cm were used for each curing condition (four for compressive strength data and two for flexural data; not included in present paper) to obtain the mean values and experimental deviations (for the sample type, see Table 4, point d).

To characterize the MKPC microstructure, total porosity and pore size distribution were analysed by Mercury Intrusion Porosimetry (MIP), while its mineralogy was determined by X-ray diffraction (XRD) at different curing ages (7, 28, 50, 70, and 90 days). MIP tests were run in specimens of 1 cm^2^ of mortar using an AutoPore IV 9500 V1.09 serial 293 porosimeter of Micromeritics Instrument Corporation (Norcross, GA, USA). The XRD tests were conducted in 80 µm powder samples using a D8 Advance Powder Diffractometer of Bruker Corporation (Billerica, MA, USA) under a step size of 0.01981° and a counting time of 0.5 s from 5° to 60° (2θ). For both techniques, the chemical reaction was stopped by immersion of the sample in isopropanol for 24 h to remove the excess of liquid water. MKPC powders from paste and mortar were used. Pore pH value and pore ion composition (PIC) were also determined according to the procedure described by Alonso et al. [24]. A sample of MKPC paste and mortar was ground to a particle size of 80 µm, and a 1:1 solid/liquid suspension using deionized water was prepared. After 5 min of stirring, the solution was filtered, and the pH was determined. Pore pH measurements were performed using a HI1043 digital electrode of Hanna Instruments (Gipuzkoa, Spain) with a pH stability range of 0 to 14. ICP-OES technique was employed to obtain the ionic composition of the pore solution (P, B, Mg, K) using a Varian 725 ES ICP Optical Emission Spectrometer of Agilent Technologies (Santa Clara, CA, USA). 

Electrochemical impedance spectroscopy (EIS) was used for the characterization of the electrical resistivity and dielectric properties of the MKPC matrices. Prismatic 1M MKPC mortar samples of 3 × 3 × 3 cm dimensions were measured over 90 days under two different moisture content: in a chamber at 100%RH and isolated in sealed plastic containers (see Table 4, point c). Two stainless steel meshes of 3 × 3 cm each were embedded in the mortars at a distance of 1.5 cm. Measurements were obtained using a sine wave AC voltage of 32 mV r.m.s at a frequency of 10 kHz using an Autolab AUT84750 potenciostat/galvanostat by Metrohm Hispania (Madrid, Spain), using an excitation potential of 10 mV in a frequency range from 10^5^ Hz to 0.01 Hz. The evolution of the electrical resistance was determined using the inflection point at different frequencies, and the electrical resistivity was calculated following Equations (5) and (6):

(5) k=S/L

(6) ρe=k ​Re

where *S* is the surface of the electrodes (*S* = 9 cm^2^), *L* is the distance between the electrodes (*L* = 1.5 cm), *k* is the constant of the cell, *R_e_* is the electrical resistance provided by the equipment, and *ρ_e_* is the electrical resistivity. Five sample replicates were prepared for each condition to use average values and to obtain the experimental error to ensure the replicability of the results. 

The pore water content was determined at different curing ages from 2 to 90 days to understand the evolution of the electrical properties with pore moisture content and curing reaction evolution. Three replicate MKPC mortar pieces for the isolated condition and two replicate MKPC mortar pieces for 100%RH condition were dried in an oven using a constant temperature of 40 °C to guarantee that the K-struvite in the MKPC matrix was not altered by the temperature [25]. The mass loss of the samples was recorded at different ages of curing for 72 h until stabilization and attributed to water mass loss.

#### 2.3.2. Aluminium Corrosion Characterization

Electrochemical measurements were carried out to monitor the corrosion response of pure Al (A1050) and AlMg (AA5754) alloy of 10 × 1.5 × 0.02 cm dimensions embedded in MKPC mortar samples. Exposure surface areas of 2 and 3cm^2^ were used for embedding in MKPC mortar systems under isolated, plastic container, and at 100%RH curing conditions, respectively. Prior to the Al introduction in the mortar system, the surface was cleaned with isopropanol to remove possible impurities. The tested metal surface was delimited using an isolating electrochemical tape. Table 5 shows the cell geometry and electrode connection for Al corrosion measurement in MKPC mortar at 100%RH and in isolated curing. Two different electrochemical cell configurations were used in this study depending on the mortar design and the moisture curing condition:

- A three-electrode cell was used for prismatic MKPC 5 × 5 × 5 cm mortars with different M/P ratios (1, 2, and 3 molar) at 100%RH (see Table 4, point a), which includes two embedded coupons of A1050 and AA5754 alloy as working electrodes, and a bar of graphite of 5 mm ∅ as counter electrode. A distance of 1 cm between working electrode and counter electrode was used. An external reference electrode of Ag/AgCl was employed.
- A three-electrode cell configuration for 9.5 × 6 cm cylinder MKPC 1M mortars under isolated curing were applied. Hermetic plastic containers covered with parafilm were employed to isolate the samples from the atmosphere and prevent evaporation (see Table 4, point b). Pure A1050 or AA5754 alloy as working electrodes and graphite as a counter electrode were used, spaced at 1.5 cm. An external Ag/AgCl electrode was employed for corrosion potential measurements (E_corr_).

A moistened sponge was placed on the mortar surface for the connection with the external reference electrode for E_corr_ measurements. In isolated conditions, an embedded plastic tube in the cement matrix was added to the cell configuration to locate the connection with the external Ag/AgCl reference electrode. The tube was kept closed between measurements to avoid drying of the MKPC matrix. E_corr_ is expressed by the standard hydrogen electrode (SHE) for the Pourbaix diagrams. Three replicate samples allowed the calculation of experimental errors.

For electrochemical measurements, an Autolab AUT84750 potentiostat/galvanostat was used and driven by NOVA 1.10.1.9 version software. In this paper, the corrosion response was carried out using the traditional method described in [26,27] of making electrical contact with the metal embedded in concrete to measure the corrosion potential (E_corr_) using an external Ag/AgCl reference electrode. The corrosion rate was determined by Linear Polarization Resistance (LPR). The polarization range used varied ±20 mV with respect to the E_corr_ [28,29]. To ensure the accuracy of the R_p_ measurements, correction for the mortar ohmic drop (IR) was carried out. The IR was determined at 10 kHz frequency and removed from the R_p_ measurement to obtain the real *R_p_* value according to [28]. *R_p_* was used to calculate the corrosion current (*I_corr_*) using Equation (7), as described in [30]:

(7) $$I_{corr}=B/R_{p}$$

**Figure 2:**
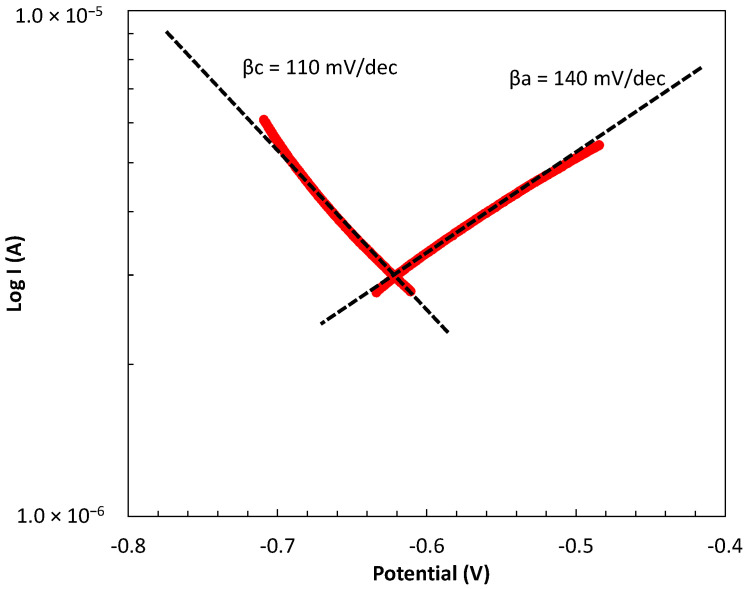
Potentiodynamic polarization curves and Tafel slopes in pure Al (A1050) embedded in 1M MKPC mortar system.

Stern–Geary constant B value of 26 was determined from the slopes of the anodic and cathodic branches in the polarization curves of pure Al (A1050), embedded in 1M MKPC mortar system for 90 days under 100%RH curing, according to Equation (8) [30]:

(8) $$B=\left(\beta a\cdot \beta c\right)/\left(2.303\cdot \left(\beta a+\beta b\right)\right)$$

where *βa* of 140 mV/dec and *βc* of 110 mV/dec were obtained, as shown in Figure 2. For this Tafel extrapolation method, the working electrode was polarized in the range of ±250 mV. This study has corroborated the selected B value of 26 mV compared with those of the literature related to aluminium corrosion, which has reported B values ranging from 26 to 29 mV [31] or 28 to 40 mV [32,33] under acidic and alkaline environments, respectively. A B value of 26 mV is also known for many actively corroding systems, especially in the case of steel embedded in cementitious systems [34,35,36]. Constant B allows us to calculate the current density (i_corr_) for pure Al (A1050) and AlMg alloys (AA5754) in all MKCP mortar matrices in present study.

After the determination of *B* and *R_p_*, corrosion rate was then calculated in µm/year using Equation (9), according to the ASTM procedure G102-89 [37]:

(9) Vcorr ​μm/year=3.27 ​· ​ ​icorr/d ​ ​· ​ ​EW

where *V_corr_* is the corrosion rate (µm/year), *i_corr_* is the current density (µA/cm^2^), *d* is the density of the metal (2.7 g/cm^3^ for Al), and *EW* is the equivalent weight (9 g/equivalent for Al).

## 3. Results

### 3.1. Characterization of MKPC Matrix Performance 

**Figure 3:**
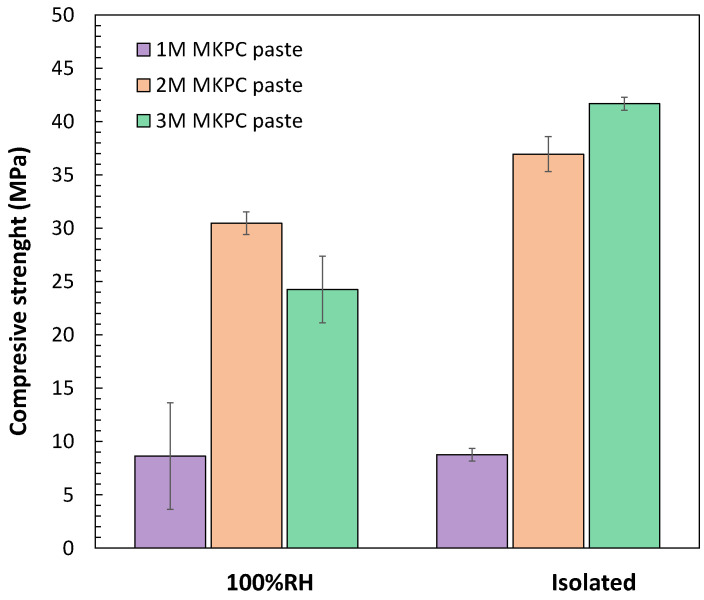
Compressive strengths and standard deviations of MKPC cement pastes at different M/P ratios (1, 2, and 3M) under 100%RH and isolated in plastic container after 28 days of curing.

To understand the effect of the M/P ratio and the moisture content on the MKPC physical stability, Figure 3 includes the mechanical strength of MKPC cement pastes after 28 days of curing. The standard deviation is also included. Under both types of curing conditions, higher M/P ratios (2 and 3M) develop more than 50% higher compressive strengths (1M: 8.6 ± 5, 2M: 35 ± 1.6 and 3M: 40 ± 0.6 MPa). The reason for this behaviour has been attributed to the pore structure evolution of the matrix during curing and to the amount of unreacted components, such as MgO, that can contribute to the densification of the matrix and to the nucleation and precipitation of reacted products. Wu et al. [38] and Wang et al. [39] suggest that at higher M/P ratios, a denser microstructure, due to the coexistence and micromorphology of the main reaction products, is generated. Wang et al. [39] suggest that at higher M/P ratios during curing time, the synthesized K-struvite crystals are fully developed, and the crystal growth is more complete, which makes the cement grains well-interconnected and form denser reinforced microstructure. On the other hand, apparently, the moisture content during curing has shown no effect with the 1M M/P ratio, while a certain increase in isolated curing for 2 and 3M was observed. The greater dispersion found with 1M at 100%RH is probably a consequence of the greater instability of this matrix that generates excess phosphates in the matrix favoured by the high moisture content in the pores, as will be discussed later in the paper.

**Figure 4:**
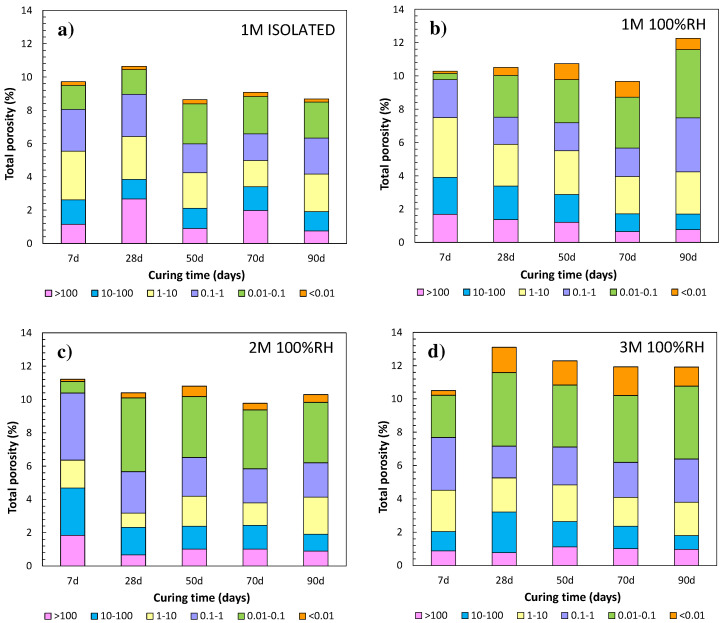
Total porosity and pore size distribution evolution in μm over 90 days of curing of MKPC mortars: (**a**) 1M isolated in plastic container, (**b**) 1M in 100%RH, (**c**) 2M in 100%RH, and (**d**) 3M in 100%RH.

To understand the differences in mechanical strength depending on the M/P ratio and the moisture content during curing, total porosity and pore size distributions were also evaluated. Pore size distribution during MKPC ageing is fundamental to understanding the pore connectivity in relation to the advance of the acid–base reaction at the microstructural level. Regarding the effect of moisture content during curing in the pore structure, total porosity and pore size distribution have been represented in Figure 4a,b. Some lower total porosities are measured during curing in isolated curing conditions, 9.3 ± 0.8%, with respect to 10.7 ± 1% at 100%RH. However, the pore size distribution shows significant differences; the proportion of capillary pores below 0.1 µm is lower in the isolated systems (2.2 ± 0.4%) than in those cured at 100%RH (3.1 ± 1.6%), with a clear increase with curing time. As suggested by Ding et al. [40], lower porosity values under isolated conditions could be explained by the presence of a larger amount of intermediate pore size (1 to 10 µm) over time, in agreement with Wu et al. [38]. The content of water in pores can have a significant effect on the evolution of reaction components in the development of MKPC microstructures. However, these differences in pore size and distribution are not appreciated in compressive strength, which are very similar but with higher scatter in 1M at 100%RH. Furthermore, in isolated curing, the pore water is consumed without any addition, being insufficient to allow the acid–base reaction to progress adequately.

The relationship between the total porosities and the pore size distribution using different M/P ratios is shown in Figure 4b–d for 1, 2, and 3M curing under 100%RH. A general trend of increase in total porosity during curing occurs with the increase in M/P ratio, with 10.0 ± 1% in 1M, 10.5 ± 0.5% in 2M, and 12.0 ± 0.9% in 3M being detected. However, these total porosities do not fit well with the higher compressive strengths at a higher M/P ratio (see Figure 3). To analyse the effect of total porosity in mechanical strength development, the pore size distribution was considered, highlighted in Figure 4 as the following pore ranges: >100, 10–100, 1–10, 0.01–0.1, and <0.01 µm. The first to notice is the increase in capillary pores of size < 0.1 µm with higher M/P ratios (1M: 3.2 ± 1.6%, 2M: 3.6 ± 1.5%, and 3M: 5.2 ± 1.3%), as also observed by [41,42,43]. Some authors [44] have detected a relationship between the decrease in porosity and the increase in compressive strengths as the M/P ratio increases from 4 to 12.

**Figure 5:**
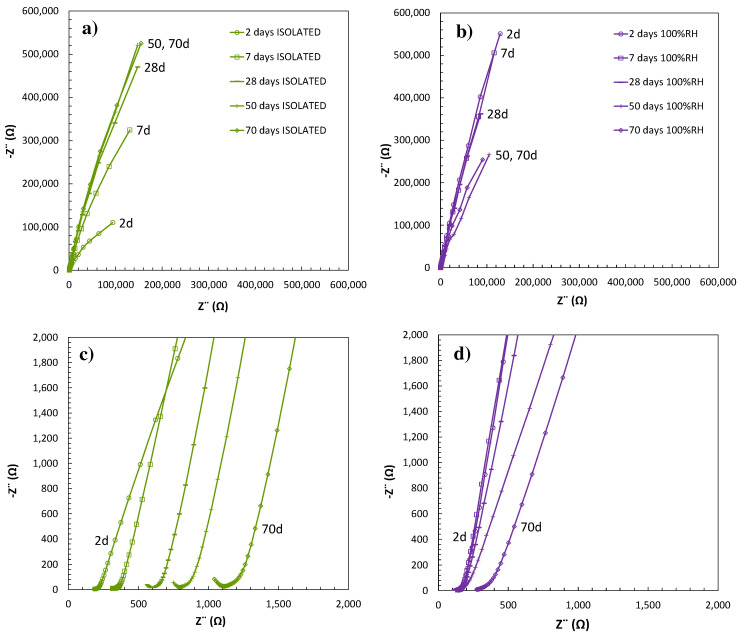
EIS response for 1M MKPC mortar: (**a**) Nyquist plots in isolated curing at low frequency, (**b**) Nyquist plots in 100%RH at low frequency, (**c**) Nyquist plots in isolated curing at high frequency, and (**d**) Nyquist plot in 100%RH at high frequency.

Synergy with the dielectric properties and pore network can also be found, as evaluated with EIS, to provide more information about the MKPC matrix at the microstructural level. Figure 5a,b show the 1M M/P EIS response under isolated and 100%RH at a time interval of up to 70 days of curing. As reported in the literature [45,46,47,48,49], the high-frequency domain in an EIS diagram is attributed to the properties of the cement matrix that can be explained in terms of the pore network: solid phase, disconnected pores, and the electrical resistance of the pore network in terms of the continuously connected pores. Zoomed areas of the Nyquist diagram are represented in Figure 5c,d, which correspond to the high-frequency domain up to 10^5^ Hz for both curing conditions. The inflection point close to the real impedance (Z’) axis has been used to determine the electrical resistance evolution with matrix maturity. Under isolated conditions (see Figure 5c), the matrix electrical resistance increases over time with a shift towards lower frequencies in the inflection point of the high-frequency domain, as also detected in Poras et al. [50]. This is attributed to the acid–base reaction progression with pore water consumption. Contrary, under 100%RH (see Figure 5d), only a slight shift in the high-frequency domain of the Nyquist diagram is observed at the end of the test without a significant increase in the mortar electrical resistance over time. This aspect has been associated with a better electrical conductivity response. Different EIS capacitive response in terms of electrochemical behaviour related to the diameter of the Nyquist semicircle is also detected at low and intermediate frequencies over time for both conditions, related to the connectivity of the pore network of the bulk matrix. A dependence of the apparent dielectric constant on the pore network was also suggested by Cabeza et al. [48], associated with the diameter of the Nyquist semicircle. Under 100%RH, a decrease in the diameter of the semicircle associated with a decrease in the dielectric constant was detected (see Figure 5b), with lower capacitive over time being related to a good pore network in the matrix. On the other hand, an increase in the diameter of the semicircle related to an increase in the capacitive matrix behaviour was observed for the isolated condition (see Figure 5a), giving poor connectivity in the pore network.

**Figure 6:**
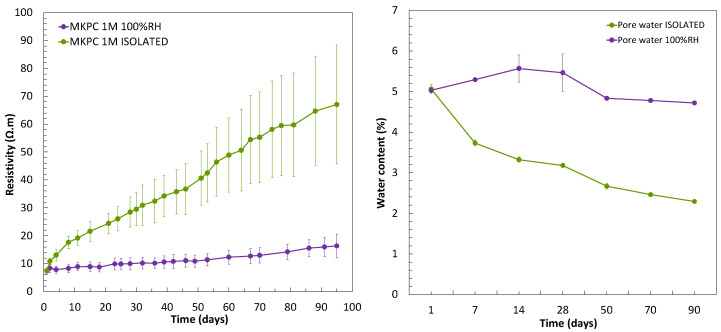
(**Left**): Electrical resistivity. (**Right**): pore water content (% bgs) evolution over curing time for 1M MKPC mortar under 100%RH and isolated curing.

Physical stability of MKPC matrices in terms of acid–base reaction progress with the time of curing was analysed through the changes in the electrical resistivity (*ρ*) calculated with the R data from EIS and the (Equations (5) and (6)) and pore water content. As observed in Figure 6 (left subfigure), average resistivity of 19.2 ± 2.7 Ω·m after 7 days and 67.1 ± 21.3 Ω·m after 90 days was measured under isolated curing. Previous studies by the authors [18] suggested electrical resistivity values of 15 Ω·m after 7 days for 1M MKPC mortars in isolated curing. The significant increase in *ρ* in isolated curing is related to a progressive decrease in the water content in the pores, as illustrated in Figure 6 (right subfigure), which is consumed in the progress of acid–base reaction. The initial water content of 5.1 ± 0.1% by g in the sample (bgs) to final values of 2.3 ± 0.05% (bgs) was determined. The increase in electrical resistivity with curing time in isolated curing could also be explained by a reduction in capillary pores (see Figure 4a) and a decrease in pore connectivity (see Figure 5a) over time, as suggested by Liu et al. [51]. In the case of 100%RH, resistivity values of 8.3 ± 1.5 Ω·m after 7 days were measured, which moderately increased over curing advance to final average *ρ* values of 16.4 ± 4.1 Ω·m. This short-change of electrical resistivity at 100%RH is a consequence of greater water content in pores, in contrast with isolated media (see Figure 6 (right subfigure)). The initial pore water content of 5.0 ± 0.01 % (bgs) to 4.7 ± 0.01% (bgs) was measured at 100%RH. A slight decrease after 28 days was identified, which could explain the small increase in resistivity values under 100%RH curing. The progression of the acid–base reaction of MgO and KH_2_PO_4_ was complete under 100%RH since a constant moisture supply was available by the environment as the reaction advanced and consumed to form more KMgPO_4_∙6H_2_O (K-struvite). Electrical resistivity data under high-moisture curing conditions are also reported in the literature [52,53,54], with values in the range of 0 to 2.5 Ω·m for MKPC cement pastes and pore solutions.

**Figure 7:**
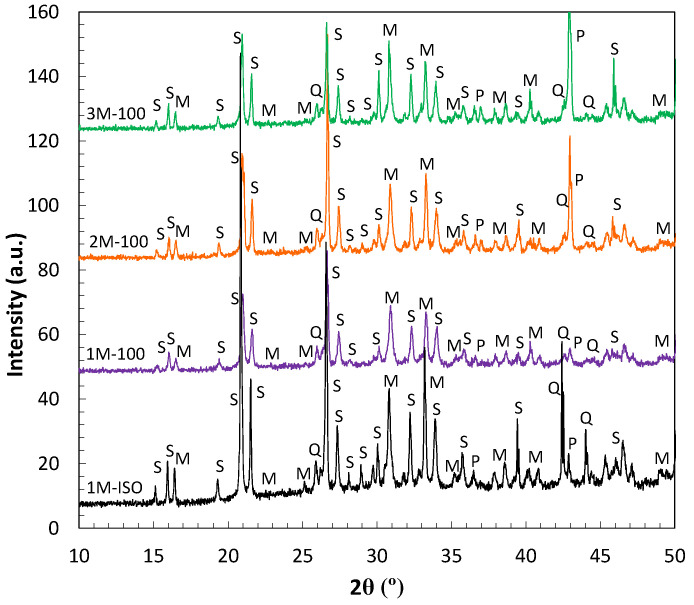
XRD pattern of MKPC mortars at different M/P ratios (1, 2, and 3M) at 100%RH and isolated curing after 28 days. Legend: S: K-struvite, P: periclase, Q: quartz and M: mullite.

**Figure 8:**
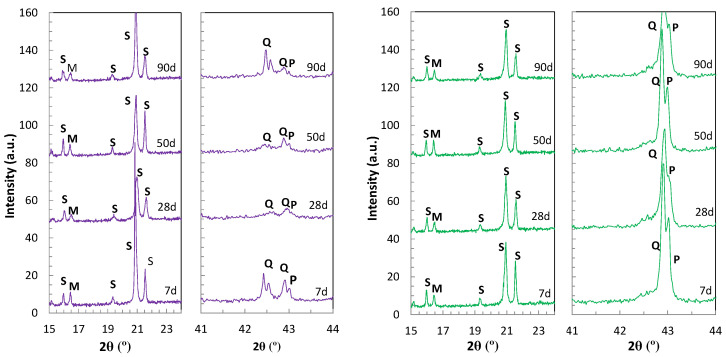
XRD patterns of MKPC mortars 1M after 90 days at 100%RH. Left: 1M M/P. Right: 3M M/P. Legend: S: K-struvite, P: periclase, Q: quartz and M: mullite.

The chemical microstructure of MKPC matrixes can be modified by the M/P ratio and the moisture content. The type of solid phases, the amount of reacted and unreacted products, and the pore solution evolution with curing are affected. Figure 7 and Figure 8 include the crystalline phases determined by XRD. Figure 7 considers the effect of the M/P ratio and moisture on the X-ray diffraction patterns. MKPC mortars at 1, 2, and 3M M/P ratios after 28 days at 100%RH curing and 1M MKPC mortar at isolated curing were used. K-struvite was detected as the main mineral phase for all systems. Diffraction reflections corresponding to quartz and mullite, coming from the FA and quart corresponding to the sand used to prepare the mortar, were also identified. As expected, at higher M/P ratios (2 and 3M), more residual periclase was detected due to more MgO and less phosphate used to prepare the different MKPC formulations (see Table 3). The coexistence of periclase and K-struvite could densify the matrix and nucleate the progressive precipitation of reacted phases in MKPC cement matrices that would contribute to the strength development, as observed in Figure 3. Also, the increase in capillary pores over time (see Figure 4) may indicate the progressive formation and nucleation of K-struvite, densifying the matrix, as suggested by Ding et al. [40]. In 1M MKPC matrices, no significant differences exist between both curing conditions. With 100%RH, the MKPC acid–base reaction is expected to be favoured, and the main reflections of K-struvite with lower residual periclase were detected at 20.99° and 42.92°, respectively, as also observed in previous studies by the authors [18]. However, the formation of other phosphate amorphous phases cannot be discarded and favoured due to the high amount of KH_2_PO_4_ in the MKPC initial formulation (see Table 3) and enough water to maintain the progress of the reaction. Under isolated curing, K-struvite and more defined reflections of non-reacted MgO were detected at 20.83° and 42.92°, respectively, probably because the water amount for the progress reaction is not enough. Other differences observed, as shown in Figure 7, were related to the intensity of the main reflection of K-struvite with 147 a.u. and 27.5 a.u. for isolated curing and 100%RH, respectively. A slight shift of 0.16° in the main reflection position of the K-struvite was observed under 100%RH, justified by a decrease in the interplanar distances by the compression of its lattice structure, as recently suggested by Li et al. [55].

The influence of the M/P ratio in the ageing of MKPC matrices was analysed in the XRD patterns for 1 and 3M M/P mortars shown in Figure 8 (till 90 days at 100%RH). Quartz and mullite, components from the FA, and sand were also identified. Figure 8 (left subfigure) shows that for 1M M/P ratio, periclase diffraction peaks are significantly reduced after 28 days of curing, indicating that most of the MgO has reacted with phosphates to form K-struvite. This is not the case with samples prepared with a 3M M/P ratio, as shown in Figure 8 (right subfigure), where intense diffraction reflections of residual periclase are identified at all curing ages. In this context, the advance of matrix ageing has been attributed to the advance of MgO and KH_2_PO_4_ acid–base reaction and the formation of K-struvite. The coexistence of both MgO and K-struvite contributes to the densification of the microstructure, with a more refined pore structure and higher compressive strength with the increase in the M/P ratio, as also suggested by [39,40]. However, the precipitation of brucite has been observed in every system, even in the systems with high periclase content, after 90 days at 100%RH.

**Figure 9:**
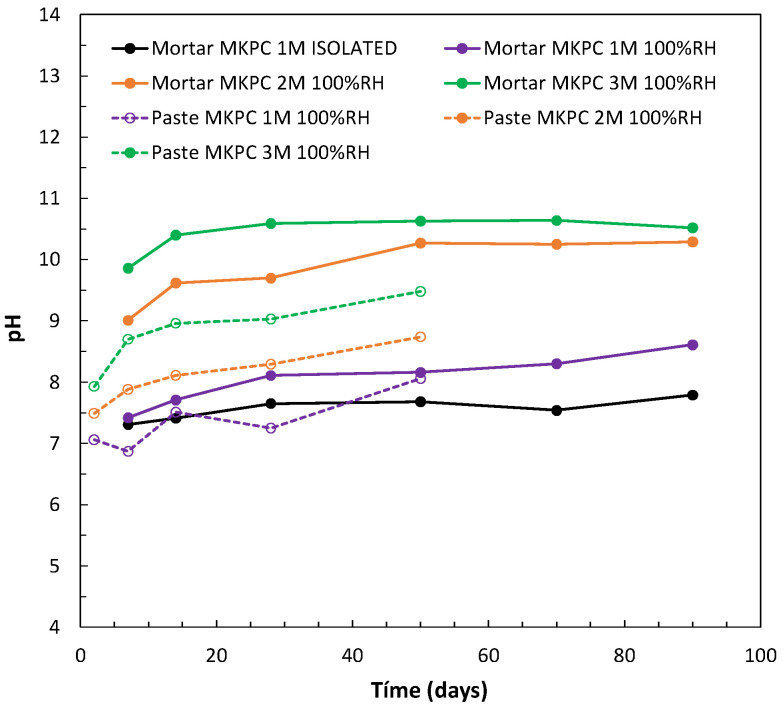
Pore pH evolution in 1, 2, and 3M M/P ratios of MKPC mortars and cement pastes at 100%RH and at isolated curing.

**Figure 10:**
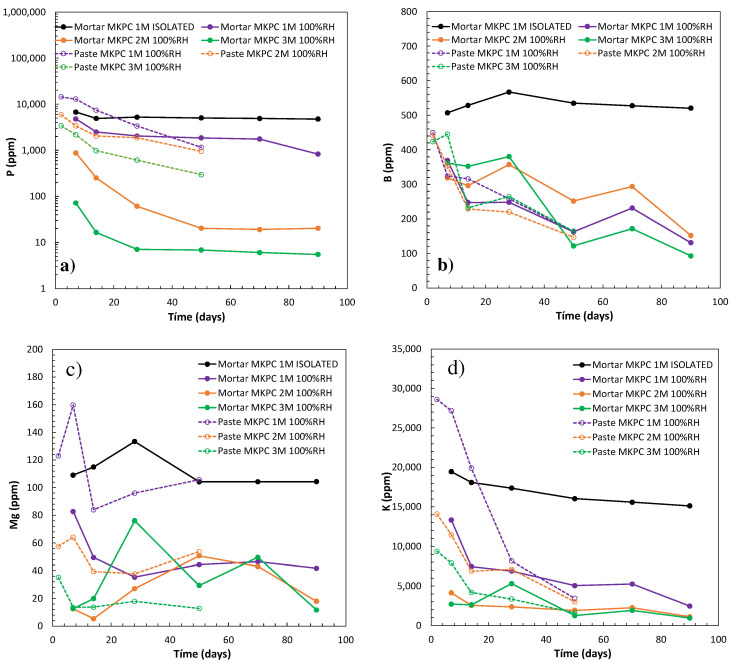
Pore ion content evolution of MKPC cement paste and mortars (1, 2, and 3M M/P) at 100%RH and isolated curing: (**a**) phosphates, P; (**b**) borates, B; (**c**) magnesium, Mg; and (**d**) potassium, K.

With regard to the chemical composition of MKPC matrixes, that is to say, the pore ion content and its evolution with the time of curing, results are compiled in Figure 9 and Figure 10. 

The pH of the liquid pore media was analysed to better understand the reaction mechanism of MgO/KH_2_PO_4_ to form the cementitious main product, K-struvite, which also affects the electrochemical stability of aluminium immobilized in the matrix. The effect of the M/P ratio (1, 2, and 3M) and the moisture content during curing (100%RH and in isolated curing) on the pore pH was determined in cement pastes and mortars, as shown in Figure 9. There was a clear increase in the pore pH values with the increase in the M/P ratio (observed in both pastes and mortars). Some authors have also predicted an increase in the pH with an increase in the M/P ratio, verified using a thermodynamic database and thermodynamic modelling [56] with pH values up to 12.1 for an M/P ratio > 1, and in [57] with a pore pH of around 10.5 for an M/P ratio of 4. Differences in pH were also found between mortars and cement paste for the same M/P ratio, the latter showing lower pH values. Mortars are initially prepared with higher w/s ratios, and these can have an impact on the solubility of the different phases and the release of the ions to the pore solution. The most plausible consequence could be associated with the equilibria with the other pore ions in the pores, such as, for instance, phosphates, which will be analysed later. An increase in the pH evolution with curing was detected for all M/P ratios and the two moisture content studied. A stabilization of pH was found after 28 days of curing, with values of 8.7 and 9.5 in cement pastes and 10.3 and 10.5 in mortars for 2M and 3M M/P ratios, respectively. Near-neutral pH values were observed at lower M/P ratios of 1M for both curing conditions. Final pH values of 8.6 and 8.1 in mortar and pastes, respectively, were identified for 1M under 100%RH, with a slight evolution over time. The isolated curing gives a lower pH of around 7.8 after stabilization. The slight difference between the two curing conditions is explained by the differences in the pore ion content related to the progress of the acid–base reaction.

The MKPC microstructural changes in the function of the M/P ratio and curing conditions are in chemical equilibria with the evolution of pore ion content, as can be deduced from Figure 10a (phosphates, P), Figure 10b (borates, B), Figure 10c (magnesium, Mg), and Figure 10d (potassium, K). Understanding the effect of the M/P ratio at 100%RH, higher phosphate content in early stages is observed at a lower M/P ratio (see Figure 10a). In addition to that, the phosphate content is higher in cement paste than in mortar, which could explain the lower pH values observed in Figure 9. This would support the fact that phosphates are also pore pH controllers. As described in previous studies [18], at pH 7, the predominant form of phosphate is H_2_PO_4_^−^ (50%) and HPO_4_^2−^ (50%), which moves to 100% HPO_4_^2−^ with higher pH [58]. In Figure 10a, a significant decrease in P occurs over time in all systems, associated with the progress of acid–base reaction of MgO and KH_2_PO_4_. K-struvite is formed as the main product, as observed in the XRD patterns (see Figure 7), corroborated by a decrease in K ions in the pore solution with time (see Figure 10d). In the case of 1M, almost all MgO reacts and soluble phosphate remains free in the pore solution in a higher proportion than in 2 and 3M. Figure 10c also shows the Mg^2+^ concentrations over time, and low contents are measured in general in the pore solution, below 80 ppm for mortar 1M and cement paste and mortar 2 and 3M. At a lower M/P ratio, some higher ion content is detected for cement paste in 100%RH and in isolated curing. Borates are also identified in Figure 10b, decreasing over time in all M/P ratios, however, no effect on the M/P ratio can be clearly derived. As suggested by Lahalle et al. [59] and Zheng et al. [60], borates are not precipitated in the crystal form as a reaction product of hardened cement matrix, at least not in crystalline form—as XRD patterns show (see Figure 7)—but remains in the pore solution. As reported by Lahalle et al. [61], the decrease in B ions in the pore solution over time could be explained by adsorption of boric acid or the precipitation of a coating layer of B(OH)_4_^−^ on the surface of MgO that could slow down its dissolution. 

To understand the effect of curing, in Figure 10, a comparison of 100%RH with isolated curing for 1M MKPC mortar is illustrated. In the isolated curing, the MKPC acid–base reaction is not complete due to a lack of external moisture supplying more phosphates, Mg, and B in the pore solution. More P ions in the isolated curing would indicate that non-reacted phosphates remained in the pore solution.

### 3.2. Characterization of Al Alloy Corrosion in MKPC Matrices 

**Figure 11:**
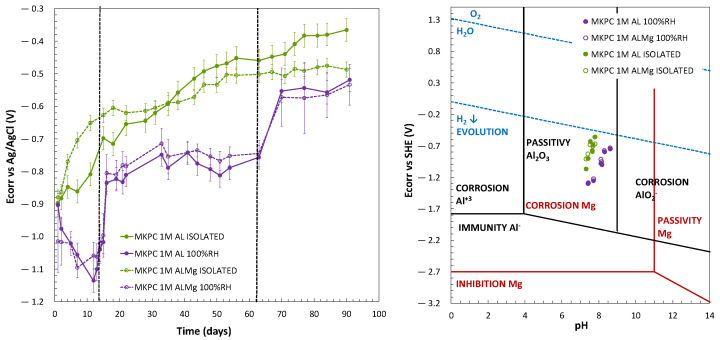
(**Left**): E_corr_ evolution (vs. Ag/AgCl). (**Right**): E_corr_ (vs. SHE) and pH for Mg and Al Pourbaix diagrams in 1M MKPC mortar systems under 100%RH and isolated curing.

It is well established that the moisture content of concrete is a significant parameter that controls the rebar corrosion phenomena [62,63]. In the present study, the effect of two levels of moisture content in the pores of MKPC cementitious matrices (100%RH and isolated curing) was evaluated to characterize the corrosion behaviour of pure Al (A1050) and AlMg 3.5% (AA5754) alloy in 1M MKPC mortars. The monitoring of corrosion potential (E_corr_) versus time is observed in Figure 11 (left subfigure). A continuous growth to more anodic values is observed for 1M MKPC mortars under isolated conditions, compared with 100%RH curing. Under isolated conditions, the acid–base reaction is not complete and leads to a lower pore pH and a higher number of phosphate ions, both effects possibly contributing to the passivation process and the formation of the passive layer, as also mentioned in [64,65]. In the first days of interaction with the matrix at isolated curing, the AlMg alloy (AA5754) shows more anodic E_corr_ values than pure Al (A1050. But this situation changes with curing time, probably due to the higher number of phosphate ions at early stages, which could be better adsorbed on the surface of the AlMg alloy (AA5754), giving a protective effect in the corrosion response. In addition to that, the pore pH may also have a significant role, as suggested by Wang et al. [20]. 

At 100%RH, three stages in the E_corr_ evolution were identified for pure Al (A1050) and AlMg alloys (AA5754). In the first stage of up to 16 days, the E_corr_ potential shows a severe evolution to the cathodic region (Al: −0.84 ± 0.03 V; AlMg: −0.81 ± 0.06 V; day 16), which after 16 days suddenly rises to a more anodic E_corr_ region till 62 days (Al: −0.76 ± 0.02 V; AlMg: −0.75 ± 0.04 V; day 62). The third region starts with a gradual evolution to more anodic E_corr_ that stabilizes at 90 days with similar E_corr_ values (Al: −0.52 ± 0.02 V; AlMg: −0.53 ± 0.06 V; day 90). It is important to notice that these three stages of the E_corr_ evolution are not detected under isolated conditions (see Figure 11, left subfigure). On the contrary, an increasing trend towards more anodic values is observed, probably as a consequence of the pore ion and lower pore pH remaining more stable, as shown in Figure 9 and Figure 10. Also, the water content is significantly reduced and consumed in the MKPC acid–base reaction progress, limiting the corrosion response due to the increase in the electrical resistivity of the matrix (as shown in Figure 6). The E_corr_ of pure Al (A1050) and the AlMg alloys (AA5754) in 1M MKPC mortar at 100%RH are very similar and approaches with exposure time to the AlMg alloy in isolated curing conditions. 

The corrosion response is corroborated by the Pourbaix diagram, drawn in Figure 11 (right subfigure) for aluminium and magnesium metals. A cross-merger of both E-pH Pourbaix diagrams was made according to [50]. Both 1M MKPC mortars (100%RH and in isolated curing) are in the Al passivity range but below the water-reduction potential with H_2_ release associated. To understand the reactivity of AlMg, the Mg Pourbaix diagram was also analysed. For both moisture contents, AlMg (AA5754) has a higher probability to locate in a corrosion stage. This effect is not appreciated in the Al Pourbaix diagram for both moisture content, where AlMg alloy (AA5754) has the same trend as pure Al (A1050), as also observed by [50]. However, as the pH of 1M MKPC at isolated curing is lower than at 100%RH, pure Al (A1050) and AlMg alloys (AA5754) presented fewer corroding risks under isolated curing. This has been associated with the adsorption of phosphate ions on the metal surface contributing to a protective effect in the corrosion response, as suggested by Wang et al. [20], as well as a lack of water to maintain the corrosion process, as demonstrated in Figure 6.

**Figure 12:**
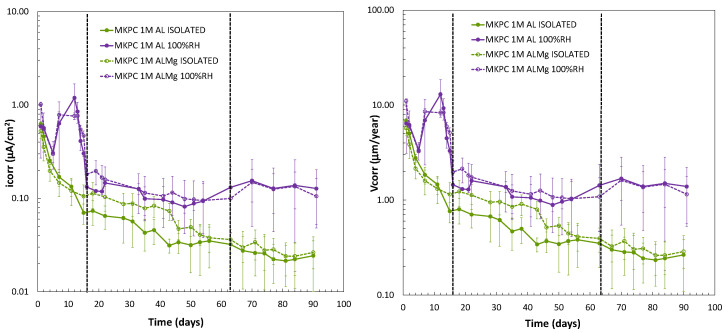
(**Left**): i_corr_. (**Right**): corrosion rate (V_corr_) versus time for pure Al (A1050) and AlMg alloys (AA5754) in 1M MKPC mortar at 100%RH and isolated curing.

Figure 12 (left subfigure) shows the i_corr_ versus time up to 90 days of test for 1M MKPC mortars evaluating the effect of moisture content on the corrosion kinetic with isolated curing and 100%RH. At 100%RH, three stages of i_corr_ evolution are also detected, as shown in Figure 11 (left subfigure). In the first stage, up to 16 days, a significant increase in the corrosion current density is detected at early stages, probably due to the high metal reactivity in the first days of interaction with the matrix, which decreases fast (Al: 0.13 ± 0.01 µA/cm^2^; AlMg: 0.18 ± 0.12 µA/cm^2^; day 16). In the second stage, up to 62 days, a decrease in the corrosion kinetic is also identified (Al: 0.13 ± 0.09 µA/cm^2^; AlMg: 0.10 ± 0.04 µA/cm^2^; day 62), which stabilizes up to 90 days in a third stage (Al: 0.13 ± 0.07 µA/cm^2^; AlMg: 0.11 ± 0.03 µA/cm^2^; day 90). Under isolated curing, a significant decrease in i_corr_ over time is observed, one order of magnitude lower than for the 100%RH condition, and the three states discussed are not as clearly visible under this endogenous (isolated) curing (Al: 0.02 ± 0.01 µA/cm^2^; AlMg: 0.03 ± 0.01 µA/cm^2^; day 90). 

To understand this evolution in the long term, the corrosion rate in µm/year was calculated using Equation (9) [37]. Under 100%RH, V_corr_ values have the same trend as i_corr_ with the three evolution stages: up to 16 days (Al: 1.45 ± 0.02 µm/year; AlMg: 1.97 ± 1.26 µm/year; at day 16), up to 62 days (Al: 1.43 ± 0.95 µm/year; AlMg: 1.09 ± 0.4 µm/year; at day 60), and up to 90 days (Al: 1.39 ± 0.82 µm/year; AlMg: 1.15 ± 0.38 µm/year; at day 90). On the other hand, under isolated conditions, a continuous decrease in the V_corr_ is detected, one order of magnitude lower than at 100%RH, with final values of 0.26 ± 0.16 µm/year and 0.29 ± 0.09 µm/year for pure Al and AlMg alloys, respectively.

**Figure 13:**
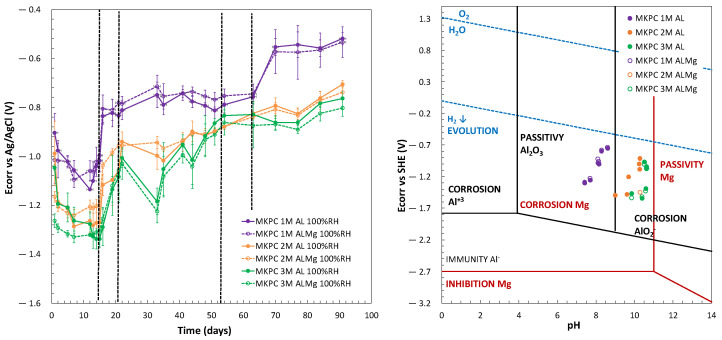
(**Left**): E_corr_ evolution (vs. Ag/AgCl). (**Right**): E_corr_ (vs. SHE) and pH for Mg and Al Pourbaix diagrams in MKPC mortars at 1, 2, and 3M M/P ratios under 100%RH conditions.

The M/P molar ratio in the MKPC formulation has been shown to be a relevant parameter in the matrix microstructure that would also affect the aluminium corrosion response as the chemical pore composition is changed (as shown in Figure 10). The monitoring of the corrosion potential (E_corr_) during 90 days of curing at a 100%RH and a 1, 2, and 3 M/P ratio is shown in Figure 13 (left subfigure). Three stages of the E_corr_ evolution are also detected in the three systems of MKPC with different levels in E_corr_ and duration, evolving from more cathodic to anodic values with the curing time, as presented in Table 6. The first stage of up to 16 days for 1M and up to 22 days for 2 and 3M was detected. The second stage was up to 62 days for 1M, 54 days for 2M, and up to 48 days for 3M. Finally, the duration of the third stage was up to 90 days in all M/P systems. As observed in Figure 13 (left subfigure), more stabilization of corrosion potential in each stage was detected for 1 and 2M, which shows a more general fluctuating evolution at a higher M/P ratio of 3M. According to this, a similar trend of evolution of pure Al (A1050) and AlMg 3.5% alloy (AA5754) was observed for all systems. A higher phosphate content in the pore solution (see Figure 10a) and lower pH (see Figure 9) in the 1M M/P ratio MKPC formulation would probably lead to less Al corrosion than at higher M/P ratios [20].

**Figure 14:**
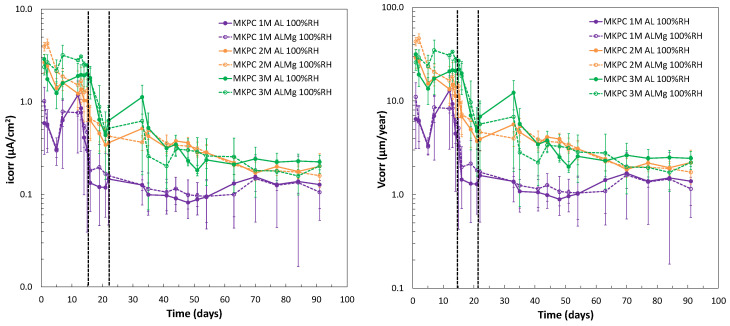
(**Left**): i_corr_ versus time. (**Right**): Corrosion rate (V_corr_) versus time for pure Al (A1050) and AlMg alloys (AA5754) in MKPC mortar system with 1, 2, and 3M M/P ratios at 100%RH.

The corrosion current density was also determined. Figure 14 (left subfigure) shows the i_corr_ versus time up to 90 days for 1, 2, and 3M M/P ratios of MKPC mortars at 100%RH, and Figure 14 (right subfigure) shows the corrosion rate (V_corr_) in µm/year. It appears that the three stages of evolution are not as clear as in the corrosion potential evolution shown in Figure 13 (left subfigure). Two defined regions in i_corr_ and V_corr_ evolution can be appreciated for all M/P ratios. The first stage, up to 16 days in 1M and up to 22 days in 2 and 3M, was identified. The second and final stages are up to 90 days, with stabilization over time observed for all systems, equalizing their corrosion response. Average values of corrosion current density and corrosion rate are summarized in Table 7 and Table 8, respectively, which could clearly evaluate the evolution of the corrosion response in the different stages and the influence of the M/P ratio. Corrosion rate values of about 7 nm/year of Al in 1M M/P ratio MKPC cement pastes were identified in the literature [17].

**Figure 15:**
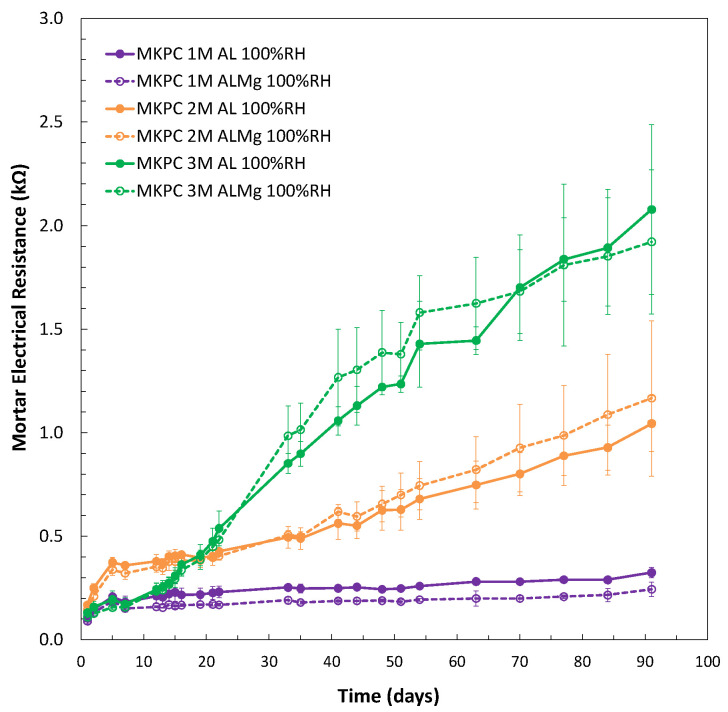
Electrical resistance (ohmic drop) versus time of MKPC mortars for 1, 2, and 3M M/P at 100%RH for pure Al (A1050) and AlMg alloy (AA5754) samples.

To understand the corrosion response evolution at 1, 2, and 3M M/P ratios, Figure 15 shows the changes during curing in the electrical resistance of the three M/P matrices obtained from the ohmic drop determination, as described for Rp measurement. A significant increase in electrical resistance was observed for a higher M/P ratio, however, higher i_corr_ values were determined (see Figure 14 (left subfigure) and Table 7). This indicates that the corrosion process is not under resistance control, but the different pore solutions should have a significant contribution to the process. The higher pore pH (see Figure 9) and the presence of lower phosphate ions in the pore solution (see Figure 10) contribute to the lower corrosion resistance of the Al alloy, as also suggested by [20]. The same behaviour for both pure Al (A1050) and AlMg alloys (AA5754) was detected.

## 4. Discussion

As described above, MKPC matrices have been considered a promising alternative to OPC cementitious matrices for the immobilization of low- to intermediate-level radioactive aluminium alloys [6,7,9,11,12,17,18,50]. OPC alkaline matrices have a pH outside of the aluminium passive domain, which is primarily responsible for the corrosion of Al alloys, limiting the formation of the passive layer, as Pourbaix suggests [9], with a high risk of hydrogen release. In the case of LILW, this situation would induce internal tensions in the steel containers of the Al alloy encapsulated. According to this, MKPC matrices with a pH range from 4 to 9 are optimum alternative cementitious materials for reactive Al alloy immobilization. The contribution of this study has been the investigation of the effect of the M/P ratio in MKPC matrices and the curing moisture content in the corrosion response of pure Al (A1050) and AlMg alloys (AA5754). The comprehensive and simultaneous analyses of the metal corrosion response and the physical–chemical microstructure change in the matrices are necessary to understand the balance for the adequate stability of the immobilization matrix and the adequate control of the corrosion process.

### 4.1. Effect of MKPC Matrix Changes and Microstructural Stability 

MKPC matrices with a low M/P ratio, as the 1M used in the present work, have derived in the appearance of chemical instabilities. The processes involved are not well understood and seem to be primarily related to the MgO and KH_2_PO_4_ dosages used in the preparation of the matrices but also can be related to the moisture at curing conditions, which can explain the different results in the literature, wherein most experiments have been performed in isolated containers.

**Figure 16:**
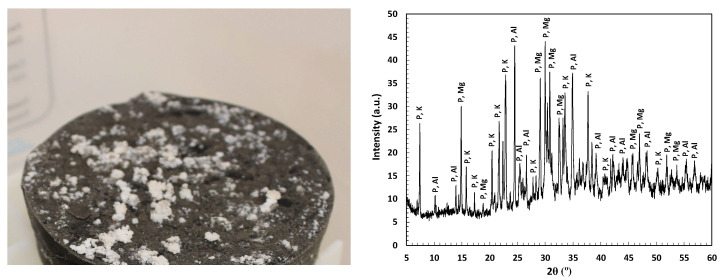
(**Left**): White efflorescences in 1M MKPC M/P ratio in 100%RH. (**Right**): XRD pattern of the white efflorescences. Legend: P-Al: aluminium phosphates, AlPO_4_; P-Mg: magnesium phosphates, H_16_MgP_2_O_10_, H_8_Mg_7_P_2_O_16_, H_7_MgPO_7_, and K-P: potassium phosphates, H_8_K_4_P_4_O_16_, H_16_K_6_P_6_O_20_.

In the present study, instability was found due to the appearance of white efflorescence in the 1M M/P ratio when cured in a high-moisture atmosphere (standard 100%RH), as illustrated in Figure 16 (left subfigure). Also, it has been found that this MKPC dosage has low mechanical strength (see Figure 3), although it has a more refined pore structure than if autogenous (isolated) curing is used. If high moisture in the matrix and the external environment is maintained and high phosphates are in the pore solution, as happens in 1M M/P ratio, the rapid dissolution of the excess of soluble phosphates can leach out and crystallize in the form of white efflorescence. The XRD analyses of the efflorescence in Figure 16 (right subfigure) indicate the consequence of precipitation of magnesium and potassium phosphates, as also postulated by Wang et al. [19], compromising the MKPC microstructural stability that needs to be guaranteed for the optimal immobilization of reactive Al waste.

To avoid these instabilities, different M/P ratios (1, 2, and 3M) and curing conditions (at isolated curing and at 100%RH) were employed in the present study, evaluating their influence on the macro and microstructure. The acid–base reaction is complete in 100%RH with the formation of K-struvite with minor residual periclase in the 1M microstructure (see Figure 7). In endogenous curing (isolated curing), the reaction does not progress properly due to the lack of water in the system, with more dissolved ions in the pore solution and lower reaction products (see Figure 10). This situation explains the lower mechanical strengths in 1M M/P ratio, and the precipitation of less stable and non-crystalline phosphate phases due to the high phosphate concentration in the pore solution cannot be discarded. At a higher M/P ratio, the coexistence of K-struvite and MgO increases the mechanical strength, also related to the K-struvite micromorphology that is fully developed at a higher M/P ratio, as suggested by Wang et al. [39].

**Figure 17:**
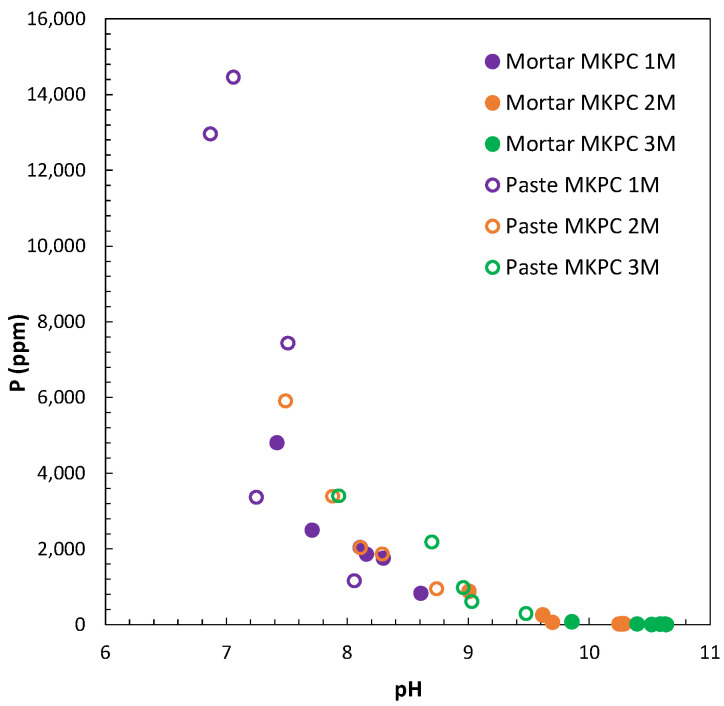
Phosphate content as function of pH for 1, 2, and 3M MKPC M/P ratios.

In addition to that, the different ion content in the pore solution should be in equilibrium and show a relationship for the different MKPC matrices; this is the case between phosphates (P) and pH, as shown in Figure 17. The content of phosphate contributes to controlling the pH in the pore solution, which increases with the increase in the M/P ratio.

### 4.2. Effect of MKPC Matrix Changes on Al Alloy Corrosion Kinetics and Risk of H_2_ Evolution 

**Figure 18:**
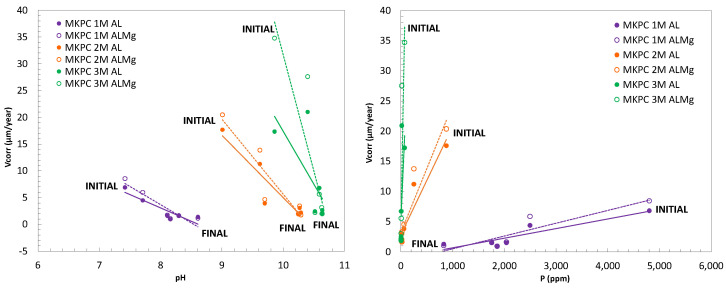
(**Left**): Corrosion rate (V_corr_) versus pore pH. (**Right**): and corrosion rate (V_corr_) versus P ion content for 1, 2, and 3M M/P ratio at 100%RH.

To better understand the contribution of pore characteristics of MKPC matrices in pure Al (A1050) and AlMg alloys (3.5% Mg) (AA5754), the corrosion response, in µm/year, was analysed with respect to the pH and phosphate (P) content in the pore solution, as shown in Figure 18. As illustrated in Figure 18, left subfigure, the changes in the pore pH of MKPC matrices have a relevant influence on the Al and AlMg corrosion response. According to the MKPC formulation, pore pH increases at a higher M/P ratio (see Figure 9), with a higher initial corrosion rate of pure Al that equalizes for all systems at the final stage. The increase in one pH unit from 7–8 (Al passive range) to 9 or 10 (for 2 and 3M) is duplicate or triplicate at the initial corrosion rate of Al alloy, from 10 µm/year to 20 and 30 µm/year. However, in the long-term interaction, even at a high pH, the corrosion rate equalizes between 3 and 5 µm/year, which suggests the positive contribution of other ions in the pore solution to control the corrosion process of Al alloys.

A significant influence on the corrosion rate of Al alloy was found with the phosphate content in Figure 18 (right subfigure). Phosphates can attach to the metal surface, generating a protective effect against corrosion, as suggested by Wang et al. [20]. The different phosphate forms can also be affected depending on the pore solution pH. The competition of H_2_PO_4_^−^ and HPO_4_^2−^ species in 1M, compared to the predominant species of HPO_4_^2−^ in the case of 2 and 3M M/P ratio, would allow the phosphates to behave as corrosion inhibitors for pure Al (A1050) and AlMg alloys (AA5754), as postulated by the authors in [18]. The borates from the boric acid are present in the pore solution, as shown in Figure 10b, as also suggested by [59,61]. The evolution of B content with curing age is independent of the M/P ratio; these borates may also contribute to the control of the corrosion of Al alloy, as suggested in [66].

Another discussion point is the influence of the M/P ratio and curing moisture condition on the volume of hydrogen released of pure Al (A1050) and AlMg at 3.5% (AA5754). This calculation is based on Faraday’s law (Equations (10)–(13)), which requires the aluminium weight loss, moles, and charge accumulated during the corrosion process, Qacum (in Coulombs), as observed in the following equations proposed by the authors:

(10) $$I_{corr}=B/R_{p}$$

(11) $$i_{corr}=I_{corr}\cdot S$$

**Figure 19:**
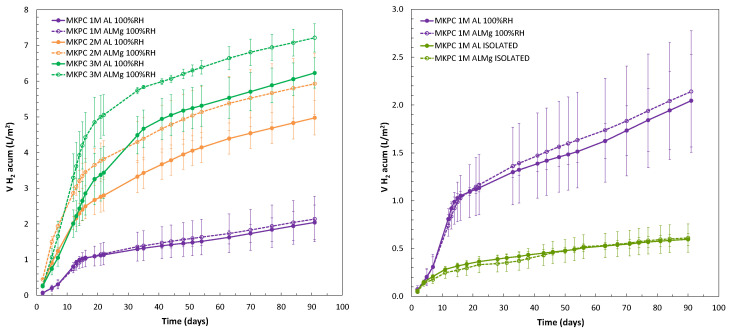
(**Left**): Estimated cumulative H_2_ release during 90 days for 1, 2, and 3M MKPC mortars at 100%RH. (**Right**): 1M MKPC under 100%RH and for isolated curing.

(12) PP ​(Al)=(Qacum ​·Mw ​Al)/(n ​·F)

(13) V ​L=32 ​· ​PP ​AlMw ​Al ​· ​(R ​· ​T)P ​

where *PP* (*Al*) is the weight loss of Al and AlMg alloys from the *i_corr_*, *S* is the exposed surface area of the metal embedded in the mortar (cm^2^), *Mw* is the aluminium molecular weight (26.98 u), *R* is the gas constant (0.082 atm L/K mol), *T* is the absolute temperature (22 ± 2 °C = 298 K), and *P* is the pressure (1 atm). Figure 19 shows the evolution of the cumulative volume of H_2_ released during the test, and the volume of H_2_ after 90 days, normalized to L/m^2^, is shown in Table 9. An increase in the volume of H_2_ release over time is detected at a higher M/P ratio, with a difference of more than two orders of magnitude between 1M and 3M. A higher initial phosphate amount in the 1M M/P ratio (see Figure 10) allows a lower amount of H_2_ release over 90 days due to the protection of the metal. 

Figure 19 (right subfigure) shows the accumulated H_2_ release in 1M MKPC mortars evaluating the effect of curing moisture conditions (isolated curing and at 100%RH). Higher H_2_ volumes are detected under 100%RH, where the phosphate ions decrease over time (see Figure 10a) due to the correct progress of the acid–base reaction under high moisture content in pores. Lower H_2_ release with constant evolution over time are found in isolated environments, which is related to higher phosphate and borate ions in the pore solution and lower pore pH (see Figure 9 and Figure 10). A similar evolution of H_2_ release for pure Al (A1050) and AlMg (AA5754) was detected at a 1M M/P ratio, higher for AlMg in 2 and 3M.

## 5. Conclusions

Each type of LILW requires optimal design and a compatible cementitious matrix to guarantee the long-term safety of the repository. MKPCs have been selected as a suitable matrix for the immobilization of radioactive aluminium waste. To optimize the efficiency of long-term immobilization of the installation, the role of MKPC dosage as a function of MgO/KH_2_PO_4_ (M/P) and moisture content in pores is considered based on the hypothesis that these two aspects affect the stability of the MKPC matrix and the corrosion kinetic of Al. Furthermore, discrepancies have been detected in the literature regarding the physical–chemical stability of MKPC matrices and the predicted hydrogen release volume. The present study allows us to detect that the instabilities are more prone at low M/P ratios and in high humidity. Corrosion kinetics and hydrogen release are greater for a high M/P ratio and high pore moisture content. A balance between both aspects is needed to ensure the long-term stability of the immobilization of Al alloy in the MKPC matrix. An M/P ratio greater than 1 is necessary to obtain the physical stability of the matrix, and an M/P ratio less than 2 does not significantly increase the corrosion kinetics and H_2_ gas release.

The specific conclusions from the present study that corroborate the beneficial use of MKPC matrices to immobilize aluminium radioactive waste can be summarized as follows.

- The corrosion process of Al alloy is affected by the pH and ion content in the pore solution. The increase in pH from 7–8 in 1M M/P to 9 and 10 in 2 and 3M M/P is duplicate and triplicate the initial corrosion rate of Al alloys that equalizes at long-term interaction.
- The presence of phosphate ions in the pore solution inhibits the corrosion process in both Al alloys. The presence of borates also has an influence on decreasing the corrosion rates. The effect of Mg in the alloy is more relevant with the increase in the M/P ratio.
- Accumulated average H_2_ release over 90 days in 1, 2, and 3M M/P ratios under 100%RH conditions were 2.04 L/m^2^ and 2.14 L/m^2^ in 1M, 4.97 L/m^2^ and 5.93 L/m^2^ in 2M, and 6.23 L/m^2^ and 7.22 L/m^2^ in 3M, for pure Al (A1050) and AlMg alloys (AA5754), respectively. With the lower moisture content in pores in the endogenous (isolated) curing, the corrosion kinetic is significantly reduced. Calculated values of H2 are 0.59 L/m^2^ and 0.61 L/m^2^ for pure Al (A1050) and AlMg alloys (AA5754).
- More of the hydrogen release occurs during the first 15 days of the interaction of the Al alloy with the MKPC matrix; values between 50–60% of the total H_2_ release at 90 days have been measured to attenuate at longer exposures and during the ageing of the matrix. This aspect has to be considered in experimental campaigns and for long-term predictions of H evolution.
- The physical–chemical stability of the MKPC matrices in terms of microstructural properties shows a high dependence on the pore moisture content and M/P molar ratio used in the formulation.
- The 1M M/P ratio exhibits lower strengths in both curing conditions, yielding matrix instabilities and phosphate efflorescences. The increase in M/P ratios allows higher capillary pores and higher mechanical strength of the matrices.
- The pore solution is mainly dominated by phosphates, borates, and K but a low Mg content. The pore ion content decreases with the M/P ratio and curing advance.

## Figures and Tables

**Table 1:** Chemical composition of pure Al (A1050) and AlMg alloys (AA5754) (in wt%).

Material	Al	Mg	Fe	Cu	Si	Mn	Cr	Zn	Ti
A1050	99.50	0.05	0.15	0.001	0.14	0.05	-	0.05	0.05
AA5754	94.50	3.50	0.40	0.30	0.40	0.30	0.25	0.20	0.15

**Table 2:** Chemical oxide composition of MgO product (% wt).

Material	Al_2_O_3_	CaO	Fe_2_O_3_	K_2_O	MgO	Na_2_O	SiO_2_	SO_3_
MgO product	0.15	0.93	0.18	0.68	97.45	0.11	0.42	0.06

**Table 3:** Formulation dosages for MKPC cement pastes and mortars for different MgO/KH_2_PO_4_ (M/P) molar ratios (1 L batch).

Material	M/P Ratio = 1M		M/P Ratio = 2M		M/P Ratio = 3M	
Compound (mass, g)	Mortar	Paste	Mortar	Paste	Mortar	Paste
MgO	70	100	110	170	140	210
KH_2_PO_4_	232	332	183	282	155	232
Sand (≤2 mm)	302	-	293	-	295	-
Fly Ash (FA)	302	432	293	452	295	442
H_3_BO_3_	6	9	6	9	6	9
Water	154	130	149	136	150	133

**Table 4:** MKPC sample geometry, type of test, curing conditions, and M/P ratio.

Type of Test	Al Alloy Corrosion		MKPC Matrix				
	E_corr_, i_corr_, V_corr_, Volume of H_2_		Electrical Resistivity and Mass Loss		Compression, MIP, XRD, Pore Ion Content and pH		
**Cement** **material**	Mortar		Mortar		Paste	Mortar	
**M/P ratio**	1, 2, 3M	1M	1M		1, 2, 3M	1M	
**Curing** **condition**	100%RH	Isolated curing	100%RH	Isolated curing	Isolated curing, 100%RH	100%RH	Isolated curing
**Sample** **Geometry cm**	5 × 5 × 5	9.5 × 6	3 × 3 × 3		1 × 1 × 6	5 × 5 × 5	9 × 2.3
	Prism	Cylinder	Prism		Prism		Cylinder
**Metal**	Al/Al-Mg		SS mesh		-		
	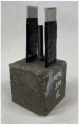	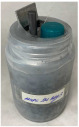	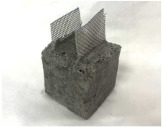		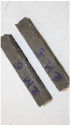	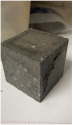	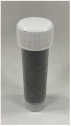
	a	b	c		d	e	f

**Table 5:** Sample cell configuration and electrode connection for electrochemical corrosion tests at 100%RH and isolated in plastic containers curing conditions.

Isolated Curing		100%RH Curing	
Cell configuration	Connection	Cell configuration	Connection
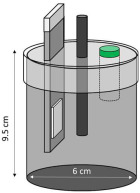	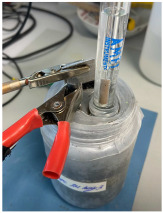	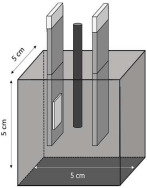	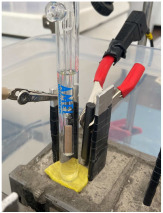

**Table 6:** E_corr_ of pure Al (A1050) and AlMg alloys (AA5754) in MKPC mortars with 1, 2, and 3M M/P ratios under 100%RH.

MKPC	M/P Ratio = 1M			M/P Ratio = 2M			M/P Ratio = 3M		
	Days	E_corr_ (V)		Days	E_corr_ (V)		Days	E_corr_ (V)	
Metal	-	Al	AlMg	-	Al	AlMg	-	Al	AlMg
1st stage	0−16	−0.84 ±0.03	−0.81 ± 0.06	0−22	−0.94 ±0.04	−0.96 ±0.02	0−22	−1.00 ±0.04	−1.03 ±0.06
2nd stage	16−62	−0.76 ±0.02	−0.75 ±0.04	22−54	−0.88 ±0.01	−0.88 ±0.01	22−48	−0.92 ±0.08	−0.93 ±0.06
3rd stage	62−90	−0.52 ±0.02	−0.53 ±0.06	54−90	−0.71 ±0.02	−0.74 ±0.02	48−90	−0.76 ±0.03	−0.80 ±0.03

**Table 7:** i_corr_ for pure Al (A1050) and AlMg alloys (AA5754) in MKPC mortars at 1, 2, and 3M M/P ratios at 100%RH.

MKPC	M/P Ratio = 1M			M/P Ratio = 2M			M/P Ratio = 3M		
	Days	i_corr_ (µA/cm^2^)		Days	i_corr_ (µA/cm^2^)		Days	i_corr_ (µA/cm^2^)	
Metal	-	Al	AlMg	-	Al	AlMg	-	Al	AlMg
1st stage	0−16	0.13 ±0.01	0.18 ±0.11	0−22	0.36 ±0.09	0.43 ±0.08	0−22	0.63 ±0.28	0.52 ±0.34
2nd stage	16−90	0.13 ±0.07	0.11 ±0.03	22−90	0.20 ±0.07	0.16 ±0.04	22−90	0.22 ±0.04	0.20 ±0.03

**Table 8:** Corrosion rate (V_corr_) of pure Al (A1050) and AlMg alloys (AA5754) in MKPC mortars with 1, 2, and 3M M/P ratios at 100%RH.

MKPC	M/P Ratio = 1M			M/P Ratio = 2M			M/P Ratio = 3M		
	Days	V_corr_ (µm/yr)		Days	V_corr_ (µm/yr)		Days	V_corr_ (µm/yr)	
Metal	-	Al	AlMg	-	Al	AlMg	-	Al	AlMg
1st stage	0−16	1.45 ±0.02	1.97 ±1.26	0−22	3.93 ±1.00	4.64 ±0.90	0−22	6.81 ±3.15	5.65 ±3.80
2nd stage	16−90	1.39 ±0.81	1.15 ±0.38	22−90	2.19 ±0.80	1.73 ±0.43	22−90	2.44 ±0.43	2.21 ±0.36

**Table 9:** Accumulated H_2_ release for pure Al (A1050) and AlMg alloys (AA5754) over 90 days at the 1, 2, and 3M M/P ratios under 100%RH and isolated in plastic containers (endogenous curing).

MKPC	M/P Ratio = 1M		M/P Ratio = 2M		M/P Ratio = 3M	
Parameter	H_2_ (L/m^2^)		H_2_ (L/m^2^)		H_2_ (L/m^2^)	
Metal	Al	AlMg	Al	AlMg	Al	AlMg
100%RH	2.05 ±0.48	2.14 ±0.63	4.97 ±0.48	5.93 ±0.86	6.23 ±0.42	7.21 ±0.39
Isolated	0.60 ±0.06	0.61 ±0.15	-	-	-	-

## Data Availability

The data presented in this study are available on request from the corresponding author.
